# Supplementary material for: Association between free thyroxine levels and clinical phenotype in first-episode psychosis: a prospective observational study
Source: PeerJ. 2023 Jun 2;11:e15347. doi: 10.7717/peerj.15347 (PMC10241168; doi:10.7717/peerj.15347)
Supplement: Supplemental Information 1 [file peerj-11-15347-s001.docx]

**Table S1. Perris and Brockington’s diagnostic criteria for cycloid psychosis**

| 1. An acute psychotic episode, unrelated to substance use or to brain organicity, with an onset between 15 and 50 years of age. 2. Sudden onset in a period of hours, or of a few days at most. 3. To arrive at a definitive diagnosis, at least four of the following symptoms should be present: 4. Some degree of confusion, ranging from perplexity to severe disorientation. 5. Mood-incongruent delusions of any kind: most often with a persecutory content. 6. Hallucinatory experiences of any kind, often related to fear of death. 7. An overwhelming, frightening experience of anxiety, not bound to particular situations or circumstances. 8. Deep feelings of happiness or ecstasy, most often of a mystical nature. 9. Akinetic or hyperkinetic motility disturbances. 10. A particular concern with death. 11. Background (oscillations of mood, but not pronounced enough to justify a diagnosis of an affective disorder). 12. There is no fixed symptomatologic combination: on the contrary, the symptomatology may change frequently during the episode. |
| --- |
