# Supplementary figures and images for: Association between free thyroxine levels and clinical phenotype in first-episode psychosis: a prospective observational study
Source: PeerJ. 2023 Jun 2;11:e15347. doi: 10.7717/peerj.15347 (PMC10241168; doi:10.7717/peerj.15347)

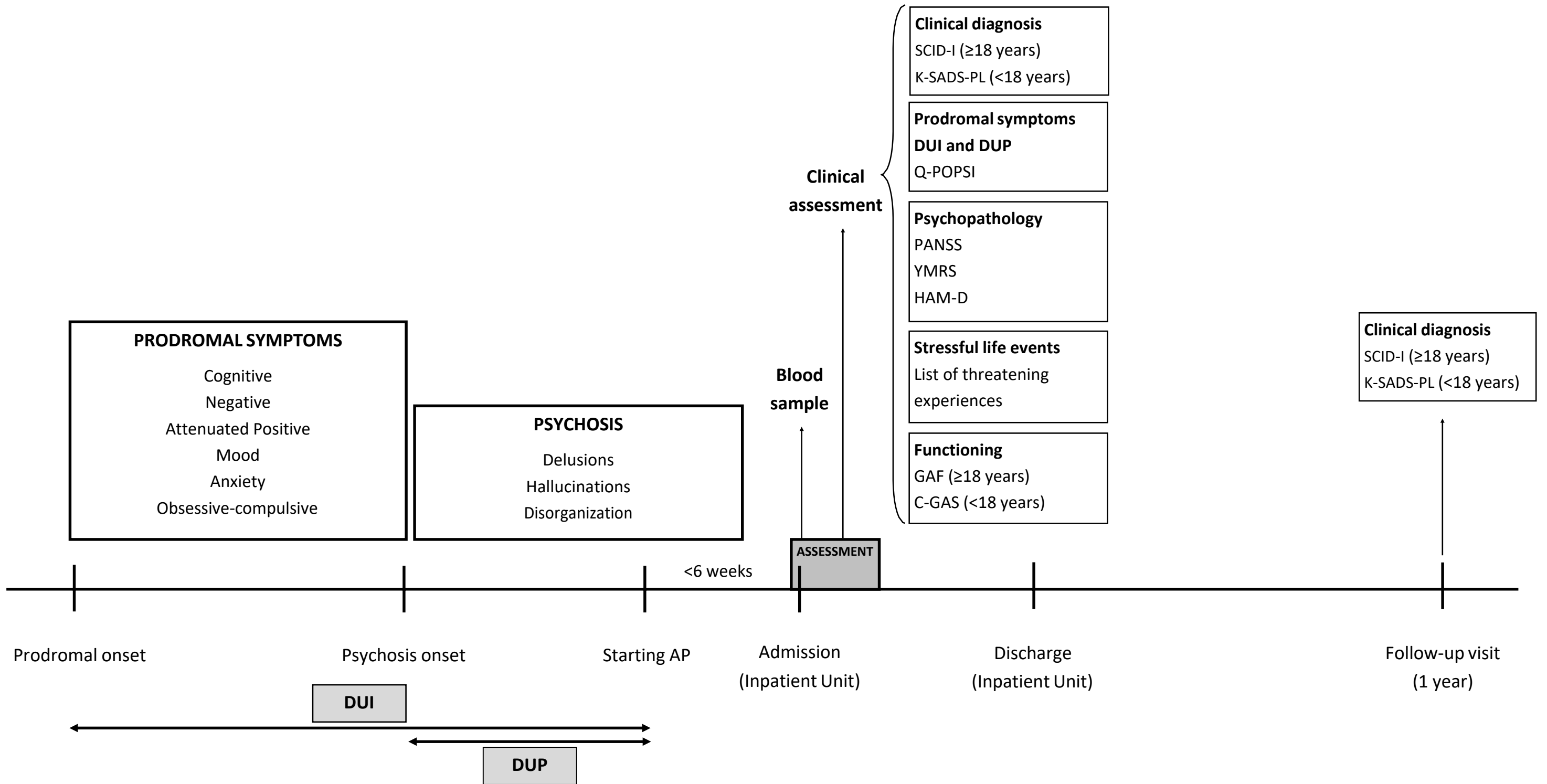

Supplement: Supplemental Information 3 [file peerj-11-15347-s003.pdf]
